# Serum Levels of VWF, t-PA, TNF-*α*, and ICAM-1 in Patients Receiving Hemocoagulase Combined with Platelet-Rich Plasma during Total Hip Replacement

**DOI:** 10.1155/2022/2766215

**Published:** 2022-01-27

**Authors:** Yaobin Huang, Bin Zhou, De Zhang, Yu Chen

**Affiliations:** Department of Joint Orthopaedics, Yuebei People's Hospital, Shaoguan City, Guangdong Province 512025, China

## Abstract

**Objectives:**

This study aimed to investigate the effect of hemocoagulase combined with platelet-rich plasma (PRP) in total hip replacement (THR) on reducing bleeding and improving knee joint function in the patients with osteoarthritis.

**Methods:**

From February 2018 to February 2020, 80 osteoarthritis patients undergoing THR were included in the study, of which 40 cases were treated with PRP and hemocoagulase (test group) in the joint capsule in THR and the other 40 cases received saline and thrombin in the joint capsule after THR (control group). Postoperative drainage and corresponding functional exercise were performed for the two groups 12 hours after operation. The outcome measures including operation time, soft-tissue release, blood routine, drainage volume, perioperative blood loss, postoperative incision inflammation, deep vein thrombosis (DVT), and range of motion (ROM) of the joint were recorded.

**Results:**

The hemoglobin and hematocrit values of the test group on the second postoperative day were significantly higher than those of the control group (*P* < 0.05). The postoperative drainage volume and perioperative blood loss were significantly lower than those of the control group (*P* < 0.05). The test group was better than the control group in the ROM of the joint at 7 and 15 days after the operation (*P* < 0.05). A lower value of prothrombin time and activated partial thromboplastin time was revealed in the test group compared with the control group (*P* < 0.05). No significant difference in the operation time, intraoperative soft-tissue release, postoperative incision inflammation, incidence of DVT, incidence of deep infection, and ROM at day 90 after THR was found in the two groups (*P* > 0.05).

**Conclusions:**

The application of hemocoagulase combined with PRP in THR can reduce perioperative blood loss, increase wound healing speed and quality, and improve coagulation and immune function. It is a safe and effective method for the patients with knee osteoarthritis who underwent THR.

## 1. Introduction

Total hip replacement (THR) is a salvage operation to alleviate the discomfort caused by osteoarthritis in the hip. THR usually leads to extensive traumatic injury and rapid bleeding, and conventional techniques cannot improve hemostasis [1]. Hemostasis plays an essential role in the surgery, especially for patients who underwent bilateral THR, which inevitably increases the amount of perioperative bleeding and allogeneic blood. Allogeneic blood transfusion is an independent risk factor affecting the prognosis of THR [2, 3]. In addition, massive bleeding will also cause coagulation dysfunction and increase the risk of infection at the surgical site [4]. Therefore, how to reduce the perioperative blood loss and allogeneic blood transfusion during THR is a huge challenge for clinicians. Platelet-rich plasma (PRP) is autologous plasma containing high concentrations of platelets above that contained in whole blood. The platelets can release a variety of growth factors with multiple regenerative properties [5]. It has been widely used in various operations, such as hair restoration [6], hip and knee osteoarthritis [7], and facial plastic surgery [8]. Enormous studies have proven that PRP contributed to reduction in blood loss and allogeneic blood transfusion [9, 10] and enhancement on wound healing [11]. In addition, PRP application in liver and spinal surgery can improve intraoperative coagulation disorders caused by autologous blood reinfusion, blood dilution, and the application of plasma substitutes [12]. Hemocoagulase is a TLE purified from snake venom [13], which was used for hemostasis in the treatment of surgical intervention. Hemocoagulase has been found to reduce bleeding effectively for the patients undergoing cardiac surgical intervention [14], breast cancer surgery [15], and fracture-related hemiarthroplasty [16]. At present, few studies on the application of PRP were found in the osteoarthritis patients undergoing THR. This article explores the effect of PRP application on perioperative blood loss and allogeneic blood transfusion in THR.

## 2. Materials and Methods

### 2.1. Study Subjects

A total of 80 patients with osteoarthritis who underwent THR in our hospital from February 2018 to February 2020 were included. The inclusion criteria were as follows: (a) no serious cardiovascular and respiratory diseases; (b) normal liver and kidney function; (c) preoperative hemoglobin (Hb) content >110 g/L, hematocrit (Hct) >35%, and normal platelet count. The exclusion criteria were as follows: (a) cardiopulmonary insufficiency and liver and kidney dysfunction; (b) long-term use of nonsteroidal anti-inflammatory drugs and less than 7 successive days of drug withdrawal before surgery; (c) history of deep vein thrombosis (DVT) or pulmonary embolism; (d) onset time of myocardial or cerebral infarction <6 months; (e) coagulation dysfunction; (f) blood system diseases. All patients were randomly divided into the PRP group (*n* = 40, preoperative platelet separation and intraoperative platelet reinfusion) and control group (*n* = 40, no performance on platelet separation and reinfusion). All the patients signed the informed consent in the study.

### 2.2. Treatment Protocols

The equipment to prepare PRP was purchased from Shandong Wei Polymer Medical Material Co., Ltd.; in brief, 60 ml of peripheral venous blood was extracted and injected into the centrifuge tube, and the PRP was prepared under aseptic conditions. The blood sample was centrifuged at 1000 r/min for 10 min, and the centrifugal radius was 13.5 cm. The blood sample was divided into 3 layers, and 24 ml of the lowest layer containing red blood cells was sucked, followed by another centrifugation on the remaining blood sample, resulting in the appearance of albuginea-like substance (platelets and white blood cells) on the surface of red blood cells. Finally, most part of plasma on the first layer was extracted, and about 12 ml of plasma remained in the centrifuge tube, which was called PRP. 2 U of hemocoagulase (Penglai Nuokang Pharmaceutical Co., Ltd., China) was diluted to 1 ml with normal saline, which was put into the syringe. The syringe containing 12 ml of PRP and the syringe containing 1 ml of diluted hemocoagulase were fixed into the special spraying tool.

### 2.3. Surgical Protocols

Venous access was performed for all the patients firstly, and the noninvasive blood pressure, oxygen saturation, and electrocardiogram were monitored. 1% lidocaine was applied to the puncture site, and radial artery puncture and catheterization were applied to monitor arterial blood pressure. All patients were anaesthetized with midazolam (0.05–0.1 mg/kg), sufentanil (1.0–2.0 *μ*g/kg), and propofol (2–2.5 mg/kg). Ammonium bromide (0.6–1.2 mg/kg) was used for induction of anesthesia. After tracheal intubation, respiratory parameters (tidal volume and respiratory rate) were adjusted according to the transcutaneous carbon dioxide (PtCO_2_) value, maintaining PtCO_2_ at 35–45 mmHg. After the patient was stable, the central venous pressure (CVP) was monitored by puncturing the right internal jugular vein under ultrasound guidance. Intraoperative continuous pumping of propofol (4–12 mg/(kg h)) and remifentanil (0.2–0.25 *μ*g/(kg·min)) was performed to maintain anesthesia. The pump injection speed was adjusted according to the intraoperative arterial blood pressure, heart rate, and EEG frequency index (maintained at 40-60) to maintain the appropriate depth of anesthesia. The control group received the same dose of saline and thrombin. The test group also adopted the hemostasis conditions of the control group. The operation time of the two groups was recorded. The choice of fresh frozen plasma was determined by the standard established by the British Committee for Standards in Haematology (BCSH) [17] and the results of the thromboelastogram (TEG). Before blood product transfusion, 10 mg of dexamethasone was given to the patients, and the blood product was heated with a blood transfusion warmer (temperature set at 39.0°C). After the operation, the patient was transferred to the anesthesia recovery room, and the tracheal intubation was removed after the patient was fully awake. After induction of anesthesia, all patients underwent ultrasound-guided bilateral iliac fascia block (0.5% ropivacaine 30 ml + dexamethasone 5 mg) and were supplemented by patient-controlled analgesia pump (sufentanil 2.5 *μ*g/kg + tropane hydrochloride) and Setron 10 mg) for postoperative analgesia. Low-molecular-weight heparin (2500 units) was routinely used for anticoagulation treatment after surgery.

### 2.4. Postoperative Treatments

Conventional heparin for the prevention of thrombosis and continuous passive joint movement were the same in the two groups. The patient's hemoglobin, hematocrit value, platelet count, prothrombin time, and activated partial thromboplastin time were recorded on the second day after surgery, and the choice of blood transfusion was determined based on routine blood examinations. The index was that hemoglobin is 35% lower than before surgery so as not to affect the incision healing and postoperative rehabilitation exercises. Postoperative drainage volume was used to calculate the total perioperative blood loss, the severity of the incision inflammatory reaction, and the incidence of deep vein thrombosis (DVT) (that is, the visual limb swelling between 7 and 14 days after surgery). Doppler ultrasonography of the lower extremities was performed to confirm whether there is DVT formation. Straight leg raising moment, the time of knee flexion up to 90°, and the ROM of the knee joint on the 7^th^, 15^th^, and 90^th^ day after surgery were observed. At 90 days after surgery, all patients were required to perform relevant examinations.

### 2.5. Statistical Processing

SPSS 22.0 software was applied to statistical analysis. The measurement data were described as mean ± standard deviation, the K-S test was used to verify their normality, and the independent sample *t*-test was used to analyze normal distribution variables. The count data were expressed as frequency or percentage and analyzed by chi-square test. *P* < 0.05 meant that the difference was significant.

## 3. Results

### 3.1. Demographic and Clinical Characteristics of Patients in the Two Groups

There were no significant differences in age, gender composition, body mass index, joint mobility, preoperative hemoglobin, hematocrit value, platelet count, prothrombin time, and activated partial thromboplastin time between the two groups of patients (*P* > 0.05, Table 1).

### 3.2. Hemocoagulase Combined with PRP Improved Anemia of the Patients Undergoing THR

The two groups showed no statistically significant difference in the operation time (*P* > 0.05). The difference in hemoglobin and hematocrit between the two groups was statistically significant (*P* < 0.05). Comparing with the control group, the test group showed a declined value of prothrombin time and activated partial thromboplastin time after surgery (*P* < 0.05, Table 2).

### 3.3. Hemocoagulase Combined with PRP Reduced Blood Loss and Accelerated Early Postoperative Joint ROM

As listed in Tables 3 and 4, no statistically significant difference was revealed in soft-tissue release between the two groups during operation (*P* > 0.05). Postoperative drainage volume and perioperative blood loss in the test group were significantly lower than those in the control group (*P* < 0.05). At 5 days after operation, there was no significant difference in postoperative incision inflammation between the two groups (*P* > 0.05). At 7 and 15 days postoperatively, the ROM of the knee joint in the test group was improved remarkably than that in the control group (*P* < 0.05), but no significant difference in joint activity between the two groups after postoperative 90 days was observed (*P* > 0.05).

### 3.4. Hemocoagulase Combined with PRP Reduced Inflammatory Response and Enhanced Coagulation Function

**Figure 1:**
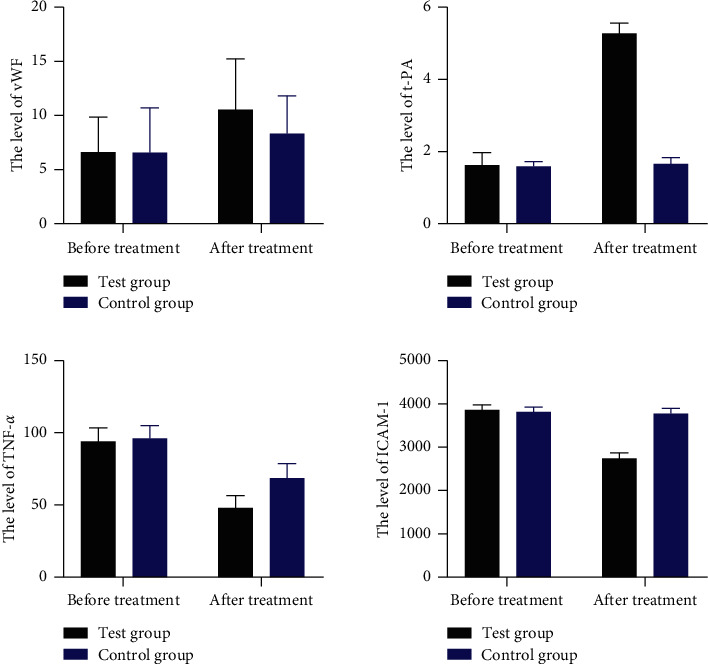
The serum levels of VWF, t-PA, TNF-*α*, and ICAM-1 between the test group and the control group before and after treatment.

Results of ELISA found that no significant difference was noted with regard to serum levels of von Willebrand factor (VWF), tissue plasminogen activator (t-PA), tumor necrosis factor-alpha (TNF-*α*), and intercellular cell adhesion molecule-1 (ICAM-1) between the test group and the control group before surgery (*P* > 0.05). After treatment, the serum level of VWF was increased, and the serum level of TNF-*α* was declined in the control group (*P* < 0.05); the serum levels of VWF and t-PA were increased, and the serum levels of TNF-*α* and ICAM-1 were declined in the test group (*P* < 0.05). It revealed that the test group exhibited higher serum levels of VWF and t-PA and lower serum levels of TNF-*α* and ICAM-1 than the control group (*P* < 0.05, Figure 1).

## 4. Discussion

It is a big challenge to stop bleeding in THR, especially massive bleeding. The increase in bleeding will inevitably lead to a decrease in platelets and coagulation factors and aggravate the bleeding tendency, which is an important factor for the increase of allogeneic blood volume during the perioperative period [18]. At present, the commonly used methods, such as endoscopic hemostasis [19], compression hemostasis [20], controlled hypotension [21], and acute normovolemic hemodilution [22], can reduce partial intraoperative blood loss and allogeneic blood transfusion in surgical interventions. However, allogeneic blood transfusion increased postoperative complication and mortality rates [23]. Autologous blood transfusion has been associated with reduced demand on allogeneic transfusion in THR [24], but autotransfusion devices impact the quality of red blood cells and remove inadequate leukocytes [25]. The effect of PRP mainly depends on the total amount of platelets obtained by separation. Increase of separated platelets is positively correlated with the platelet count in blood after transfusion, which can achieve early hemostasis. The concentration of platelets and other factors in PRP is 5–10 times higher than that of conventional autotransfusion, which can effectively avoid the loss of platelets and fibrinogen, coagulation factor V, coagulation factor VIII, and other coagulation components [26], and PRP has been confirmed as a promising and safe method for the musculoskeletal diseases, which repairs tissues with poor healing capacity [27]. It has been PRP which can maintain the function of platelets without reducing the level of hemoglobin and restore coagulation function during the perioperative period [28, 29]. Everts et al. [30] and Davies et al. [31] found that PRP can significantly reduce blood loss after total knee arthroplasty. However, some reports indicated no significant difference in controlling blood loss using PRP [32, 33]. Ekback et al. [34] found that PRP combined with autologous blood and preoperative red blood cells can significantly reduce blood loss and allogeneic blood requirements for total hip replacement surgery, increase the number of platelets and P-selectin after surgery, and promote early wound healing. This study found that PRP can significantly reduce perioperative blood loss and postoperative drainage volume.

TEG continuously and dynamically monitors the whole blood coagulation process, including thrombosis and fibrinolysis. TEG data include fibrinogen, platelets, and hemoglobin, and the results are closer to the occurrence and development of blood coagulation in the body than traditional methods [35]. The activated clotting time is a sensitive indicator reflecting the endogenous coagulation pathway. Reaction time (R) reflects the activity and function of the coagulation factors. Coagulation time (K) reflects the rate at which the blood clot strength reaches a certain level, which is affected by coagulation factors and fibrinogen [36]. In this study, the two groups showed increased prothrombin time and activated partial thromboplastin time after surgery than those before surgery, and the patients who received hemocoagulase combined with PRP revealed a significantly lower value of these two biomarkers compared to the patients who underwent saline and thrombin. In addition, a significantly higher hemoglobin and hematocrit value was indicated in the test group in relation to the control group after operation. These outcomes suggested that hemocoagulase combined with PRP improved anemia and coagulation function disorder of the osteoarthritis patients who underwent THR. Furthermore, ELISA results demonstrated that, comparing to the control group, the test group showed an increased level of VWF and t-PA after treatment. VWF is a large adhesive polymer protein involved in hemostasis, and its activity represents the functionality of proteins. VWF deficiency can lead to von Willebrand disease and bleeding [37]. t-PA is a serine protease that activates plasminogen only when it is combined with fibrin, and it is released into blood from the vascular endothelium during trauma or stress [38]. It is involved in the regulation of cell adhesion or migration, wound healing, angiogenesis, and tumor cell metastasis [39]. This study also found that declined expression of TNF-*α* and ICAM-1 was shown in the group that received PRP. TNF-*α* is an inflammatory cytokine in vivo activated by macrophages and monocytes and participates in inflammatory response and immune response [40]. Inhibition expression of TNF-*α* contributed to the therapy of breast cancer [41]. ICAM-1, also known as CD54, is a member of the immunoglobulin superfamily (IgSF). It is an important adhesion molecule mediating adhesion reaction [42]. These results in the study suggested that PRP can improve wound healing and inflammatory response, as well as coagulation function. At postoperative 7 and 15 days, the patients who received PRP showed better ROM of the joint than the other group, which indicated that PRP enhanced wound healing and joint function.

In summary, this study shows that the application of PRP technology in THR can significantly reduce perioperative blood loss and postoperative drainage volume and enhance wound healing and immune function. PRP application has a significant protective effect on the coagulation system. However, it may have a greater impact on the patient's hemodynamics due to the blood collection before the PRP technique. Therefore, perioperative hemodynamic monitoring must be strengthened. In addition, large samples need to be enrolled in further study to verify these presented outcomes.

## Figures and Tables

**Table 1:** Preoperative basic information between the two groups.

Item	Test group	Control group	*t*-value	*P* value
Age	68.9 ± 8.1	68.5 ± 8.2	1.15	0.25
Gender (male/female)	10/30	11/29	0.56	0.36
BMI	30.1 ± 1.8	29.8 ± 1.9	0.34	0.27
Hemoglobin (g/L)	111.3 ± 10.8	111.9 ± 10.5	0.27	0.09
Hematocrit value (%)	41.2 ± 1.9	40.8 ± 1.8	0.35	0.27
Prothrombin time (s)	12.0 ± 1.6	12.4 ± 1.5	0.58	0.13
Activated partial thromboplastin time (s)	33.5 ± 5.1	32.9 ± 4.8	0.15	0.07

**Table 2:** The operation time and postoperative observation indexes after operation between the two groups.

Item	Test group	Control group	*t*-value	*P* value
Operation time	88.9 ± 5.6	89.1 ± 5.8	1.32	0.36
Hemoglobin (g/L)	78.3 ± 9.2	68.2 ± 8.1	2.89	0.02
Hematocrit value (%)	36.2 ± 2.1	31.5 ± 2.3	2.36	0.03
Prothrombin time (s)	13.1 ± 0.3	14.4 ± 1.4	5.74	<0.01
Activated partial thromboplastin time (s)	34.5 ± 4.6	37.8 ± 5.3	2.97	<0.01

**Table 3:** Intraoperative and postoperative outcome measures of the two groups.

	Control group (*n*)	Test group (*n*)
Soft-tissue release	25	26
Wound inflammatory reactions	15	8
Deep infection	2	0
Hip DVT	5	4

DVT: deep vein thrombosis.

**Table 4:** Joint function assessment and blood loss of the two groups.

Group	Postoperative drainage (ml)	Total blood loss (ml)	ROM of the joint at 7 d after surgery (°)	ROM of the joint at 15 d after surgery (°)	ROM of the joint at 90 d after surgery (°)
Test group	206 ± 24	553 ± 120	15 ± 12	23 ± 18	40 ± 25
Control group	416 ± 35	796 ± 132	10 ± 15	16 ± 15	38 ± 26
*t*-value	5.46	3.56	2.58	3.25	1.32
*P* value	0.02	0.03	0.01	0.01	0.25

## Data Availability

The data used to support the findings of this study are included within the article.

## References

[1] Jonas J., Tomas V., Broz T., Durila M. (2020). Utility of rotational thromboelastometry in total hip replacement revision surgery (case-control study). *Medicine (Baltimore)*.

[2] Carroll K., Dowsey M., Choong P., Peel T. (2014). Risk factors for superficial wound complications in hip and knee arthroplasty. *Clinical Microbiology and Infections*.

[3] Newman E. T., Watters T. S., Lewis J. S., Jennings J. M., Wellman S. S., Attarian D. E. (2014). Impact of perioperative allogeneic and autologous blood transfusion on acute wound infection following total knee and total hip arthroplasty. *The Journal of Bone and Joint Surgery*.

[4] Kwong L. M., Turpie A. G. G., Tamayo S., Peacock W. F., Yuan Z., Sicignano N. (2017). A post-marketing assessment of major bleeding in total hip and total knee replacement surgery patients receiving rivaroxaban. *Current Medical Research and Opinion*.

[5] Shahid M., Kundra R. (2017). Platelet-rich plasma (PRP) for knee disorders. *EFORT Open Reviews*.

[6] Justicz N., Derakhshan A., Chen J. X., Lee L. N. (2020). Platelet-rich plasma for hair restoration. *Facial Plastic Surgery Clinics of North America*.

[7] Bennell K. L., Hunter D. J., Paterson K. L. (2017). Platelet-rich plasma for the management of hip and knee osteoarthritis. *Current Rheumatology Reports*.

[8] Peng G. L. (2019). Platelet-rich plasma for skin rejuvenation: facts, fiction, and pearls for practice. *Facial Plastic Surgery Clinics of North America*.

[9] Carless P. A., Rubens F. D., Anthony D. M., O’Connell D., Henry D. A. (2011). Platelet-rich-plasmapheresis for minimising peri-operative allogeneic blood transfusion. *The Cochrane Database of Systematic Reviews*.

[10] Ma J., Sun J., Guo W., Li Z., Wang B., Wang W. (2017). The effect of platelet-rich plasma on reducing blood loss after total knee arthroplasty: a systematic review and meta-analysis. *Medicine (Baltimore)*.

[11] Yao D., Feng G., Zhao F., Hao D. (2021). Effects of platelet-rich plasma on the healing of sternal wounds: a meta-analysis. *Wound Repair and Regeneration*.

[12] Safwat A. M., Reitan J. A., Benson D. (1997). Management of Jehovah’s witness patients for scoliosis surgery: the use of platelet and plasmapheresis. *Journal of Clinical Anesthesia*.

[13] Waheed H., Moin S. F., Choudhary M. I. (2017). Snake venom: from deadly toxins to life-saving therapeutics. *Current Medicinal Chemistry*.

[14] Yao Y. T., Yuan X., Fang N. X. (2019). Hemocoagulase reduces postoperative bleeding and blood transfusion in cardiac surgical patients: a PRISMA-compliant systematic review and meta-analysis. *Medicine (Baltimore)*.

[15] Lu X., Yang X., Zhu M., Hua B., Niu X., Xiao W. (2017). Hemostatic effect of hemocoagulase Agkistrodon on surgical wound in breast cancer surgery. *Zhongguo Yi Xue Ke Xue Yuan Xue Bao*.

[16] Qiu M., Zhang X., Cai H., Xu Z., Lin H. (2017). The impact of hemocoagulase for improvement of coagulation and reduction of bleeding in fracture-related hip hemiarthroplasty geriatric patients: a prospective, single-blinded, randomized, controlled study. *Injury*.

[17] Levi M., Toh C. H., Thachil J., Watson H. G. (2009). Guidelines for the diagnosis and management of disseminated intravascular coagulation. British Committee for Standards in Haematology. *British Journal of Haematology*.

[18] Saito K., Kaiho Y., Tamii T., Nakamura T., Kameyama E., Yamauchi M. (2019). Intraoperative hemorrhage in revision total hip arthroplasty: a retrospective single-center study. *Journal of Anesthesia*.

[19] Brock A. S., Rockey D. C. (2015). Mechanical hemostasis techniques in nonvariceal upper gastrointestinal bleeding. *Gastrointestinal Endoscopy Clinics of North America*.

[20] Roghani-Dehkordi F., Zangeneh E., Kermani-Alghoraishi M. (2021). Manual versus mechanical compression hemostasis approach after coronary angiography via snuffbox access. *The Anatolian Journal of Cardiology*.

[21] Kosucu M., Tugcugil E., Arslan E., Omur S., Livaoglu M. (2020). Effects of perioperative magnesium sulfate with controlled hypotension on intraoperative bleeding and postoperative ecchymosis and edema in open rhinoplasty. *American Journal of Otolaryngology*.

[22] Crescini W. M., Muralidaran A., Shen I., LeBlan A., You J., Giacomuzzi C. (2018). The use of acute normovolemic hemodilution in paediatric cardiac surgery. *Acta Anaesthesiologica Scandinavica*.

[23] Inoue Y., Ishii M., Fujii K., Kitada K., Kuramoto T., Takano Y. (2021). The effects of allogeneic blood transfusion in hepatic resection. *The American Surgeon*.

[24] Pawaskar A., Salunke A. A., Kekatpure A., Chen Y., Nambi G. I., Tan J. (2017). Do autologous blood transfusion systems reduce allogeneic blood transfusion in total knee arthroplasty?. *Knee Surgery, Sports Traumatology, Arthroscopy*.

[25] Serrick C. J., Scholz M., Melo A., Singh O., Noel D. (2003). Quality of red blood cells using autotransfusion devices: a comparative analysis. *Journal of Extra-Corporeal Technology*.

[26] Xu J., Gou L., Zhang P., Li H., Qiu S. (2020). Platelet-rich plasma and regenerative dentistry. *Australian Dental Journal*.

[27] Everts P., Onishi K., Jayaram P., Lana J. F., Mautner K. (2020). Platelet-rich plasma: new performance understandings and therapeutic considerations in 2020. *International Journal of Molecular Sciences*.

[28] Ekback G., Edlund B., Smolowicz A., Axelsson K., Kjellberg J., Carlsson O. (2002). The effects of platelet apheresis in total hip replacement surgery on platelet activation. *Acta Anaesthesiologica Scandinavica*.

[29] Giordano G. F., Rivers S. L., Chung G. K., Mammana R. B., Marco J. D., Raczkowski A. R. (1988). Autologous platelet-rich plasma in cardiac surgery: effect on intraoperative and postoperative transfusion requirements. *The Annals of Thoracic Surgery*.

[30] Everts P. A., Devilee R. J., Brown Mahoney C., Eeftinck-Schattenkerk M., Box H. A., Knape J. T. (2006). Platelet gel and fibrin sealant reduce allogeneic blood transfusions in total knee arthroplasty. *Acta Anaesthesiologica Scandinavica*.

[31] Davies G. G., Wells D. G., Mabee T. M., Sadler R., Melling N. J. (1992). Platelet-leukocyte plasmapheresis attenuates the deleterious effects of cardiopulmonary bypass. *The Annals of Thoracic Surgery*.

[32] Guerreiro J. P., Danieli M. V., Queiroz A. O., Deffune E., Ferreira R. R. (2015). Platelet-rich plasma (PRP) applied during total knee arthroplasty. *Revista Brasileira de Ortopedia*.

[33] Horstmann W. G., Slappendel R., van Hellemondt G. G., Wymenga A. W., Jack N., Everts P. A. (2011). Autologous platelet gel in total knee arthroplasty: a prospective randomized study. *Knee Surgery, Sports Traumatology, Arthroscopy*.

[34] Ekback G., Ryttberg L., Axelsson K., Christianssen F., Kjellberg J., Carlsson P. (2000). Preoperative platelet-rich plasmapheresis and hemodilution with an autotransfusion device in total hip replacement surgery. *Journal of Clinical Apheresis*.

[35] Sorensen B., Ingerslev J. (2005). Tailoring haemostatic treatment to patient requirements-an update on monitoring haemostatic response using thrombelastography. *Haemophilia*.

[36] Horton S., Augustin S. (2013). Activated clotting time (ACT). *Methods in Molecular Biology*.

[37] Favaloro E. J., Henry B. M., Lippi G. (2021). Increased VWF and decreased ADAMTS-13 in COVID-19: creating a milieu for (Micro) thrombosis. *Seminars in Thrombosis and Hemostasis*.

[38] Madhani J., Movsowitz H., Kotler M. N. (1993). Tissue plasminogen activator (t-PA). *Therapeutic Drug Monitoring*.

[39] Malgorzewicz S., Skrzypczak-Jankun E., Jankun J. (2013). Plasminogen activator inhibitor-1 in kidney pathology (review). *International Journal of Molecular Medicine*.

[40] Idriss H. T., Naismith J. H. (2000). TNF alpha and the TNF receptor superfamily: structure-function relationship(s). *Microscopy Research and Technique*.

[41] Cruceriu D., Baldasici O., Balacescu O., Berindan-Neagoe I. (2020). The dual role of tumor necrosis factor-alpha (TNF-alpha) in breast cancer: molecular insights and therapeutic approaches. *Cellular Oncology*.

[42] Bui T. M., Wiesolek H. L., Sumagin R. (2020). ICAM-1: a master regulator of cellular responses in inflammation, injury resolution, and tumorigenesis. *Journal of Leukocyte Biology*.

